# The Promising Potential of Menstrual Stem Cells for Antenatal Diagnosis and Cell Therapy

**DOI:** 10.3389/fimmu.2014.00205

**Published:** 2014-05-19

**Authors:** Maroun Khoury, Francisca Alcayaga-Miranda, Sebastián E. Illanes, Fernando E. Figueroa

**Affiliations:** ^1^Laboratory of Nano-Regenerative Medicine, Faculty of Medicine, Universidad de Los Andes, Santiago, Chile; ^2^Cells for Cells, Santiago, Chile; ^3^REGENERO, Consortium in Tissue Engineering, Santiago, Chile

**Keywords:** menstrual stem cells, stem cells, menstrual blood, cell therapy, mesenchymal stem cells

## Abstract

Menstrual-derived stem cells (MenSCs) are a new source of mesenchymal stem cells isolated from the menstrual fluid. Currently, there is a growing interest in their clinical potential due to fact that they are multipotent, highly proliferative, and easy to obtain in a non-invasive manner. Sampling can be repeated periodically in a simplified and reproducible manner devoid of complications that no existing cell source can match. MenSCs are also free of ethical dilemmas, and display novel properties with regard to presently known adult derived stem cells. This review details their distinctive biological properties regarding immunophenotype and function, proliferation rate, differentiation potential, and paracrine effects mediated by secreted factors. Their possible role in antenatal diagnosis is also discussed. While more insight on their immunomodulatory and diagnostic properties is needed, the impact of clinical and epidemiological factors, such as age, use of contraceptives, or hormonal status still requires further investigations to properly assess their current and future use in clinical application and diagnosis.

## Introduction

**Figure 1:**
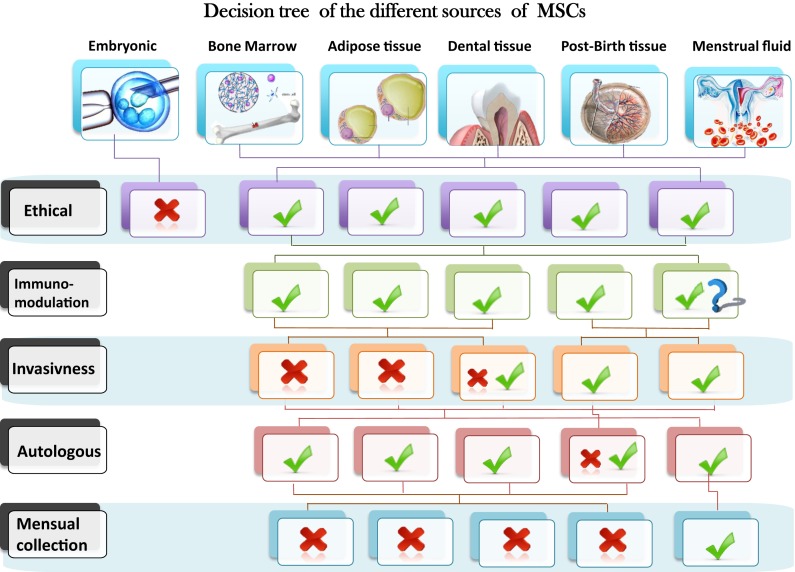
**Schematic representation of a decision tree based on six sources of MSCs and five different criteria related to their availability, isolation procedure and different properties**.

Mesenchymal stem cells (MSCs) are pluripotent progenitor cells with self-renewing capacity and potential ability of differentiating into various specialized cell types under specific conditions. Adult stem cells are derived from different sources, such as bone marrow, adipose tissue (AD), or post-natal tissues such as umbilical cords and placenta. MSC have recently received a great deal of attention because of their therapeutic potential for treating immune mediated or neoplasic human diseases. However, the difficulty of isolating adult stem cells from diverse tissues due to the invasiveness of the extraction methods and the need for *in vitro* expansion are limiting points in their clinical applications. Therefore, many studies have focused on the search for novel stem cells that can be effectively used for therapeutic purposes without these limitations. While each clinical application will have its own selection criteria for choosing the most appropriate MSCs source, a representation of a decision tree based on six sources of MSCs and five different criteria related to their availability, isolation procedure, and different properties is presented in Figure 1.

A study published in 2007 identified and characterized a new source of stem cells within the menstrual fluid. They showed that menstrual-derived stem cells (MenSCs) are a highly proliferative stem cell population that is able to differentiate under standard laboratory conditions into specific-tissue cells of three germ layers (1). These cells present a good alternative to MSCs present in other sources such as bone marrow, adipose, and post-birth tissues due to the fact that they have higher proliferation rates and are of easy access with no need for surgical procedures or hospitalization, a feature that none of the existing sources can match. They are also free of ethical dilemmas and display novel properties with regard to the presently known adult derived stem cells.

## Are MenSCs Just Another MSCs Source?

A detailed characterization of the MenSCs is a pre-requisite for a head-to-head comparison with related cells from other sources. This will pave the way for evaluating possible advantages of MenSCs and also their safety/efficacy profile for clinical applications.

### Proliferation, senescence, and migration

Meng et al. showed that MenSCs from the menstrual fluid of young healthy women grew at a rate of one doubling every 19.4 h, which is twice faster than bone marrow-derived MSCs (BM-MSCs), estimated at 40–45 h in early passages (1). In an effort to understand such a high proliferation rate, one should look back at their origin and physiological function. The endometrium consists of the epithelial layer and the underlying lamina propria. This layer is structurally and functionally divided into the functionalis – with glands extending from the surface epithelium – and the lower basalis (2). The upper two-thirds of the functionalis are shed during menstruation and are a major part of the collected menstrual fluid. Recent studies have provided ample evidence for the existence of stem/progenitor cells in human endometrium. Human uterine endometrial cells were once established as a feeder layer to maintain the undifferentiated state of human embryonic stem cells, since the high expression of embryotrophic factors and extracellular matrices plays a vital role in their growth (3). Human endometrium thus contains a population of stem cells responsible for this remarkable regenerative ability, and menstrual fluid include a population of such cells that can be expanded in culture and still remain able to express the phenotype of multiple lineages.

A good proliferation rate is essential for clinical applications since cell-based therapies are dose dependent, preferably with cells from lower passages. In most human trials, one million/kg is the dose of choice; however, when allogenic or repeated usage seems possible, escalating the yield of cultures becomes of utmost importance. Nonetheless, a high proliferation is also a two-edged sword that could lead to genetic instability or the exhaustion of a specific stem cell pool. In fact, these MenSCs have been largely expanded *in vitro* without any mutation or visible abnormality at the chromosomal level reported so far. They maintained a telomerase activity greater than 50% even at passage (P) 12 compared with human embryonic stem cells (4), and also appear to mildly express the chemokine receptor CXCR4 and the respective receptor for stromal cell-derived factor-1 (SDF-1), which play a significant role in the mediation of MSC migration (5). More interestingly, in our hands these cells did not show any sign of stem cell exhaustion evidenced by a steady expression of stromal stem markers, a stable proliferation rate, and colony-forming-unit (CFU) potential when comparing early (P3) versus old (P12) passages (unpublished data). Such a high proliferative rate in the face of genetic stability, with apparent preservation of multipotency, indicates this new type of stem cell could present unexpected therapeutic properties, a fact that is also implied by their extensive differentiation capabilities.

### Immunophenotype

MenSCs have been shown to be positive for mesenchymal stem cell markers including CD9, CD29, CD105, and CD73, and negative – as expected – for hematopoietic markers such as CD34, CD45, and CD133 (6). However, some groups have reported positive expression of embryonic markers such as SSEA-4 and Nanog in MenSCs that were not found on MSCs from other sources (7–10). This raises the question whether these cells presenting earlier markers of stemness represent a more primitive progenitor than MSCs from other sources. Nonetheless, a second group of researchers showed a different pattern of expression in cells isolated and cultured under comparable conditions (1). In Table 1, we list an exhaustive comparison of published phenotyping profiles from all available published studies. In our Lab, we have further characterized these cells, not only for mesenchymal and embryonic markers, but also for endothelial and epithelial traits, as other cell types might represent a source of contamination of the MenSCs culture. These quality-control parameters are essential when comparing similar cells from different sources.

**Table 1:** **Comparison of the different immunophenotypic profile of MenSCs**.

Markers	Cellular expression	Meng et al. (1)	Borlongan et al. (11)	Patel et al. (4)	Allickson et al. (8)	Cui et al. (9)	Khanjani et al. (12)	Mou et al. (10)
CD14	Myelomonocyte	(−)				(−)		
CD34	Hematopoietic progenitor and stem cell, endothelial cell	(−)		(−)	(−)	(−)		(−)
CD38	Variable levels on hematopoietic and no-hematopoietic cell	(−)		(−)				
CD45	Leukocyte	(−)		(−)	(−)	(−)	(−)	(−)
CD133	HSC/endothelial progenitor cell	(−)		(−)		(−)	(−)	
STRO-1	MSC	(−)						
SSEA-4	ESC	(−)		(+)	(+)			
Nanog	ESC	(−)	(+)					
CD9	MSC, hematopoietic, and epithelial cell	(+)		(+)				
CD29	MSC	(+)		(+)	(+)	(+)		(+)
CD73	B and T-lymphocyte, MSC, endothelial cell	(+)				(+)		(+)
CD41a	Megakaryocyte and platelet, found in MSC	(+)						
CD44	MSC, hematopoietic cell (except platelet)	(+)		(+)	(+)	(+)		
CD90	Hematopoietic, MSC, T-lymphocyte	(+)		(+)	(+)	(+)		(+)
CD105	MSC, vascular endothelial cell, myeloid, and lymphoid leukocyte	(+)		(+)	(+)	(+)	(+)	(+)
OCT-4	ESC	(+)	(+)		(+)		(+)	
CXCR4	Stem cell chemotaxis		(+)	(+)				
CD166	MSC, activated leukocyte			(+)	(+)		(+)	
CD49f	ESC			(+)				
MHC I	All cells (except erythrocyte)			(+)		(+)		
MHC II	APC			(−)		(−)		(−)
LIN				(−)				
CD117 (c-kit)	HSC/Germ cell			(+)	(+)	(−)		
CD13	Myelomonocyte, endometrial stromal cell, MSC					(+)		
CD54	MSC					(+)		
CD55	MSC, hematopoietic cell					(+)		
CD31	Endothelial cell					(−)		
CD50	Leukocyte, endothelial cell					(−)		
CD106	Macrophage, endothelial cell						(−)	
Vimentin	MSC						(+)	

*Abbreviations: MSCs, mesenchymal stem cells; APC, antigen-presenting cells; ESC, embryonic stem cells; HSC, hematopoietic stem cells*.

### Differentiation potential and regenerative properties

The ability of MenSCs to differentiate into adipose, bone, cartilage, cardiac, neural, hepatic, and pancreatic cell types has been shown using standard differentiation techniques and media. A study by Hida et al. using coculture with fetal mouse cardiomyocytes evidenced immortalization mediated by human telomerase reverse transcriptase (hTERT) on MenSCs (13). They also demonstrated spontaneous beating upon cardiogenic differentiation. When their cardiac differentiation potential in a scaffold culture system differentiated, MenSCs exhibited higher expression of cardiac marker (TNNT2) when compared with induced BM-MSCs (14).

In addition, these multipotent cells had the ability to differentiate into respiratory epithelial cells, neurocytes, myocytes, endothelial cells, pancreatic cells, hepatocytes, adipocytes, and osteocytes (1). A recent paper, showed a comparable hepatic differentiation ability of MenSCs with bone marrow-derived MSCs (BM-MSCs) through the expression of many hepatic markers such as albumin (ALB), cytokeratin 18 (CK-18), and tyrosine aminotransferase suggesting this new source as a safe alternative to BM-MSCs for cell-based therapies in chronic liver diseases (12). Furthermore, the differentiation potential of MenSCs into glial lineage was compared with bone marrow-stem cells (BMSCs), where both sources showed up regulation of glial fibrillary acidic protein, Olig-2, and MBP and down regulation of Nestin protein (15). Li et al. showed that MenSCs present a new source for the generation of induced pluripotent stem cells (IPS) with a high reprograming efficiency (16).

However, it is important to note that these experiments were conducted exclusively *in vitro*, and that differentiation status was determined only phenotypically using specific antibodies (1). A thorough investigation needs to be undertaken to validate these claims *in vivo* and show that the differentiated cells possess as well functional properties.

### Immunomodulation properties

Mesenchymal stem cells exert extensive immunomodulatory effects *in vitro* and *in vivo*, since they have been shown to inhibit mixed lymphocyte reaction (MLR), promote regulatory T cell generation (Tregs) (17), and to curb T helper (Th) 1 and Th17 differentiation among other suppressive effects. The fact they remain hypoimmunogenic or immune privileged has allowed their successful therapeutical use even in allo or xenogenic conditions. However, their action seems somewhat complex, since they have been shown to abrogate or conversely to exacerbate different (or even the same) autoimmune disease model under varying experimental conditions (18). While extensive progress has been made to decipher the immune features of MSCs, the description of the functional and immune effects of MenSCs is still only in its initial stages. Bearing in mind the similarity, but also the differences displayed by the MSCs, we isolated from menstrual fluid – as opposed to bone marrow – a simple extrapolation of functional or regenerative properties seems unwarranted. More so since the exploration of specific functional properties and safety issues are considered a pre-requisite to reach clinical application.

Of note, the endometrium is known to be an integral part of the mucosal immune system. It seems uniquely poised to initiate antigen specific effector as well as immunosuppressive actions, leading to responses that are protective from infectious pathogens while preserving the integrity of the fetus (19). It is therefore not unexpected that these newly discovered stem cells might exert potent immune mediated effects. Nonetheless, there is an understandable dearth of clinical or even pre-clinical data at the present time, given the recent identification of these cells. Zhong et al. reported the feasibility of allogeneic transplantation of MenSCs into four compassionate cases of patients with multiple sclerosis, where no related side effects were found after a year of follow-up, though no immune function studies were reported (20). In fact, we are not aware of any description of the use of MenSC in autoimmune human or animal models of disease (7). However, in a preliminary report of beneficial effects in a murine model of critical limb ischemia, MenSCs were shown to suppress lymphocyte MLR and the production of interferon gamma (IFN-γ) and tumor necrosis alpha (TNF-α) in a dose dependent manner *in vitro* (21).

The complete assessment of the effects of MenSCs on lymphocyte proliferation and alloreactivity in a contact dependent and contact independent manner in transwell experiments, in comparison with BM-MSCs is required to fully unravel their immunomodulatory effect. One published report indicates that MenSCs would exert opposite effects on the MLR response at different target: MenSCs ratios (22). This emphasizes the need for further studies providing insight into the mechanisms involved in this potentially new cell therapy-based application. This includes the evaluation of immunostimulatory molecules such as MHC I and II, CD40, and CD80/86. While BM-MSCs have been described to express antigen presenting (MHC I and even low level MHC II) in response to IFN-γ, they still remain immune privileged since they do not express co-stimulatory (CD80/86) molecules that are required to shift the immune response from a tolerogenic to an effector phenotype (23). Indeed, the main effect of IFN-γ on MSCs is the final “licensing” or activation of their immunosuppressive and reparative properties that tend to occur mainly in the presence of tissue damage. Thus, IFN-γ, concomitant with TNF-α or other proinflammatory cytokines (IL-1α or IL-1β) or mitogens (LPS), triggers a cascade of cellular events responsible for many of the immunosuppressive effects of MSCs both *in vitro* and also *in vivo* (24–27). These entail the upregulation of several chemokines (i.e., CCL-2/MCP-1), adhesion molecules (VLA-4, VCAM, and the SDF-CXCR4 axis among others), and of inducible nitric oxide synthase (iNOS) in the case of murine MSCs. Lymphocytes then migrate into the proximity of MSCs, where T cells are suppressed by nitric oxide (NO) (27). In the case of human MSCs, suppression appears to be exerted by exhaustion of tryptophane, mediated by indoleamine dehydrogenase (IDO) instead of NO (28). In addition, non-contact dependent factors also contribute to the immune effects of BM-MSCs, including prostaglandin E2 (PGE2), IL-6, IL-10, Galectin-1, and TNF-α-induced protein 6 (TSG-6) (29). These broader or even the species specific mechanisms have not yet been analyzed in the case of MenSCs.

Furthermore, and in an effort to understand the contrasting clinical effect reported for MSCs in mouse models of human rheumatoid arthritis (18, 30) and SLE (31, 32), our group has recently evaluated the role of BM-MSCs in the differentiation of Th1, Treg, and Th17 cells (33, 34). The balance or dysregulation of these CD4^+^ helper subpopulations is a critical factor governing disease pathogenesis and clinical response in several immune mediated diseases including murine and human SLE (35). Finding a possible explanation for the disparate clinical results of cell therapy, we initially described that MSCs suppressed Th17 cells under resting conditions, but surprisingly, expanded them once activated (33). In further transwell experiments, we evidenced the need for cell contact to suppress Th17 proinflammatory cell function (34). This methodology is currently under investigation in our group, for the full evaluation of Th1/Th17/Treg modulating properties of MenSCs, which are presently unknown.

### The secretome as “cell-free” therapy

The potency in tissue restoration mediated by paracrine factors of a broad range of bioactive molecules (secretome) produced by MSCs has raised interest in further exploring this aspect for potential therapeutic applications. This mechanism includes various main actions: immunomodulation, anti-apoptosis, angiogenesis (36), and support of the growth and differentiation of local stem and progenitor cells, and chemoattraction (37, 38). This secretion of factors or secretome could be exploited to extend the therapeutic possibilities of MSCs for treatment of a variety of diseases. The administration of MSC-released factors or conditioned medium (CM), could avoid some of the limiting factors associated with cell therapy such as immune incompatibility, tumorigenicity, costs, and waiting time for *ex vivo* expansion. This would provide an alternative option with affordable costs, excellent quality-control, consistency, and reproducibility. A wide range of different growth factors, cytokines, and extracellular matrix proteins (ECM) have been identified as constituents of the *in vitro* cultured MSC secretome. Additionally, several reports also showed that MSCs are able to secrete large amounts of micro and nanovesicles such as exosomes (39). The exosomes, released by most cells, are potent mediators of cell–cell communication due their ability to transfer proteins, lipids, and functional genetic material such as mRNA and miRNA (40, 41). Exosomes are released from cells constitutively, or following activation that significantly increases their secretion. To date the best MSCs characterized secreted proteins are those released by umbilical cord MSCs (UC-MSC) (42), AD (43), and BM-MSC (44). Several authors have documented that cells increase the liberation of vesicles in response to different types of stresses, such as hypoxia, acidosis, oxidative stress, thermal stress, and cytotoxic drugs (45). Since MenSCs niche, homeostasis and physiological condition are different from the sources mentioned above, one can speculate that they might possess a specific secretome signature that will differentiate them from MSCs found in other environmental condition.

For example, the necessary activity against pathogens in the endometrium could condition their secretome, probably through the release of antimicrobial factors. Krasnodembskaya et al. determined in a pneumonia mouse model that in response to stimulation by *Escherichia coli* inhibiting bacterial growth (46).

At the paracrine level, little is known regarding the factors secreted by MenSCs. Meng et al. described that MenSCs secrete matrix metalloproteinases (MMP3 and MMP10), cytokine growth factors [granulocyte macrophage colony-stimulating factor, GM-CSF; platelet-derived growth factor (PDGF)-BB] and angiogenic factors (angiopoietin-2, ANG-2) *in vitro*, in quantities 10–200,000 times higher than UC derived cells (1). However, no difference was observed with others angiogenic factors like VEGF, HGF, and EGF. While the regenerative and therapeutic potential of MenSCs-conditioned media have not been fully evaluated in an animal model yet, a study of an *in vitro* stroke model of oxygen glucose deprivation (OGD) determined that OGD-exposed primary rat neurons that were co-cultured with MenSCs or exposed to MenSCs-conditioned medium (MenSCs-CM) exhibited a significant decrease in cell death (11). It has been recently shown that MensCs can reverse hyperglycemia in diabetic mice most probably through paracrine factors since human insulin-producing cells was not detected in the pancreas of the injected mice (47).

In our hands, MenSCs showed a stronger supportive potential for hematopoietic stem cell (HSC) cultures, than BM-MSCs under cell-to-cell contact conditions (submitted data). We also showed that the non-contact condition (transwell) resulted in the CD34^+^CD133^+^ HSCs expansion although it was lower than that of the direct cell interactions with the stromal cells. These results suggest that MenSCs might display a quantitative and/or qualitatively distinct “secretome,” or panel of surface molecules capable of exerting distinct contact and paracrine effects on their targets. Furthermore, their protein expression profile can also be modified through the overexpression of factors of interest as they were shown to be permissive for retroviral transduction (48).

Taken together, these studies suggest that MenSCs share some properties with other MSCs but might functionally produce factors that are specific to them. This can be investigated through a comparative analysis of their secretome under different stimulation conditions, including a profiling of their exosome content.

## Safety Concerns and Clinical Applications

From a safety perspective, concerns have emerged around the procedure of collecting sterile samples, as under many countries regulations, cell and tissue collection and storage must be done in sterile conditions. This has been circumvented by a pre-treatment of the collected sample with antibiotics prior to culture, and by working in a sterile area under good manufacturing practice (GMP) conditions with proper product release criteria. Another concern is the development of endometriosis and the possibility of activation or progression of dormant tumors. To address this aspect, we performed a chronic tumorigenicity and toxicity studies, where progressive doses from 1 to 10^6^ MenSCs were injected subcutaneously in both male and female immunocompromised NOD/SCID il2rγ null mice. No sign of tumor development or toxicity was detected after a 16 weeks follow-up (unpublished data). In a different experimental setting (12), injected 2 × 10^6^ MenSC in nude mice (12). According to the histological examination, no evidence of tumor growth was found in inoculation site and the examined tissues had no morphological characteristics of tumor as judged by H & E staining. Moreover, to assess whether MenSCs modulate tumor growth, a rat glioma model was used. The injected cells showed a substantial inhibition of the tumor growth when compared to the control group (49).

The first report of clinical usage of MenSCs involved the allogenic injection of four patients with Multiple sclerosis, with a total dose of 16–30 million cells. Treated patients showed no apparent physical or serological abnormalities at follow-up (20). More recently, Medistem, a stem cell company, launched a phase II clinical trial with MenSCs, planning to enroll a total of 60 patients with congestive heart failure, receiving escalating doses up to 200 million cells from a universal donor. According to the published report in 2013, 17 patients have been injected with no treatment associated adverse events reported (50). Medistem has also obtained FDA clearance to begin Phase I trials in the US for treatment of critical limb ischemia, an advanced form of peripheral artery disease.

In all the MenSCs studies mentioned in this review, cells were isolated from healthy donors. There are no published reports yet characterizing the property changes of MenSCs isolated from epidemiologically different background donors. Thus the effects of age, hormonal status (post-puberty versus pre-menopausal), or prior contraceptive usage remain unexplored. Since stem cells are sensitive to environmental changes and stress conditions, one can only speculate if these variations might affect their function and properties. While it is known that proliferation and therapeutic potential are greatly impacted by the pathological conditions of the donors, little is known on the extent of the effect of these physiological changes on MenSCs. An epidemiological study comparing the secretome, phenotype, and immunomodulatory among other properties would present a valuable guide for the formulation of inclusion and exclusion criteria of donors for a stem cell-based therapy.

## MenSCs as a Diagnostic Tool?

As MSCs properties are modulated by environment factors, it also becomes important to analyze the role of these changes in pathological conditions.

Of the 130 million newborns each year, 8 million die before their first birthday. A contributing factor in many of these deaths is poor pregnancy outcome as a result of a complication of pregnancy, including hypertensive syndromes (e.g., pre-eclampsia – PE); poor fetal growth (e.g., intra-uterine fetal growth restriction – IUGR); gestational diabetes and preterm birth. Each occurs with an incidence of 5–10% and are responsible for the majority of obstetric and pediatric morbidity and mortality and can permanently impact on lifelong health. As an example, PE has become one of the main causes of maternal and fetal morbidity and mortality in the world, and has also been strongly associated with an increased risk of later-life death due to cardiovascular disease, independent from other risk factors (51–53). On the other hand, over the past 15 years, much has been discussed and published about the profound effects that sub-optimal health conditions during pregnancy, especially during early stages, have on the pre-disposition of the newborn to adult diseases (i.e., developmental origins of disease paradigm). Therefore, the understanding of the early processes during implantation and early stage embryo development, will not only impact on the outcome of contemporaneous pregnancies (including, early pregnancy loss, pre-eclampsia, intra-uterine growth restriction, pre-term birth, gestational diabetes, and maternal death) but also on newborn morbidity and mortality and their susceptibility. These evidence highlights the need of accurate diagnosis of the pre-disposition to, or early detection of disease during pregnancy, or even before that, allowing the implementation of effective treatments to prevent the occurrence of the disease.

It is now clear that the physiopathological process of many pregnancy diseases begins with an inadequate trophoblast invasion early in pregnancy (54). Several hypotheses have been proposed to explain the abnormal trophoblastic invasion early in pregnancy, e.g., PE or IUGR, many of them suggesting that it might be triggered by an altered maternal immune response or a defective development of maternal tolerance to the allogeneic fetus. Epidemiological evidence supporting this idea has been published by many groups, suggesting the importance of the maternal immune system in the pathogenesis of placental originated diseases. Different studies have been performed to characterize the local and systemic immune milieu of these patients as an explanation for the abnormalities of placentation observed in PE (55–57). Normal pregnancy is considered as a (T helper) Th2 type immunological state that favors an immunosuppressive environment in order to prevent fetal rejection (58). Since, MSCs have been widely implicated in immunosuppressive mechanisms targeting a range of target cells, in the context of antenatal screening, one area of great interest is to identify if MenSCs are also implicated in these complications. This could be achieved through a comparative study of the changes in their immunomodulatory and paracrine factors in comparison to MenSCs isolated from donors with uncomplicated pregnancy history.

Recent data, suggest that microvesicles (MV) are released from the placenta and their concentration in maternal plasma increases during normal pregnancy (59, 60). They contain placenta-specific proteins and miRNA and, as such, may be differentiated from maternally derived MV (61). The concentration of exosomes has been reported to increase in association with pre-eclampsia and we have also established that MVs release is changed when placental cells are exposed to different environment (submitted data). Moreover, we have been able to demonstrate that the content, proteins, and miRNA. Therefore, complications of pregnancy that affect placental perfusion or exposure to abnormal concentrations of factors that modulate the release of MVs will be reflected in their concentration and cargo in the maternal blood. It has been shown that MSCs are among cells that produce high amount of MVs (39), with a known therapeutical effect in myocardial ischemia injury (62), liver fibrosis (63), and other diseases (64, 65). Since abnormal concentration and content of placental-derived MVs in maternal blood is a surrogate measure of placental dysfunction, it will be of great interest to analyze the content of MVs isolated specifically from MenSCs and study their predictive biomarker properties in the diseases and whether they can be established as an early diagnostic test in pre-symptomatic patients.

## Conclusion

Although MenSCs have been tested only in very limited disease models, these cells have been shown to possess various regenerative properties under physiological and pathological conditions. From a translational point of view, MenSCs appear to have practical and also biological advantages over other stem cell sources. While some clinical research group and companies launched clinical trials using these cell, these fast developments in the face of lacking data, underscore the need to characterize the differentiation potential and immunological properties of well defined populations of MenSCs. The need for this type of information is decisive with respect to the development of safe and effective cell therapies for clinical application in human diseases.

The other to be investigated property of MenSCs is their potential as biomarkers that could be highly informative of the risk of asymptomatic early pregnant women subsequently developing complications of pregnancy. Such tests will offer valuable clinical information that will provide an opportunity for timely and appropriate intervention.

Future research and new evidence would greatly contribute to propulse MenSCs to the top list of best proven source of MSCs for new therapies and novel diagnostic tools.

## Conflict of Interest Statement

Maroun Khoury is the CSO of Cells for Cells and REGENERO, Francisca Alcayaga-Miranda received research funds from Cells for Cells. The other authors declare no conflicts of interest.
